# Overview of Major Classes of Plant-Derived Anticancer Drugs

**Published:** 2009-03

**Authors:** Amr Amin, Hala Gali-Muhtasib, Matthias Ocker, Regine Schneider-Stock

**Affiliations:** 1 *Department of Biology, UAE University, U.A.E.;*; 2 *Department of Biology, American University Beirut, Lebanon;*; 3 *Department of Medicine 1, University Hospital Erlangen, Germany;*; 4 *Department of Pathology, Otto-von-Guericke University Magdeburg, Germany;*; 5 *Experimental Tumorpathology, Department of Pathology, University Erlangen, Universitätsstr. 22, Erlangen, Germany*

**Keywords:** EGCG, thymoquinone, paclitaxel, pomiferin, sulforaphane, cancer

## Abstract

Cancer is the second leading cause of death worldwide. Conventional cancer therapies cause serious side effects and, at best, merely extend the patient’s lifespan by a few years. Cancer control may therefore benefit from the potential that resides in alternative therapies. The demand to utilize alternative concepts or approaches to the treatment of cancer is therefore escalating. There is compelling evidence from epidemiological and experimental studies that highlight the importance of compounds derived from plants “phytochemicals” to reduce the risk of colon cancer and inhibit the development and spread of tumors in experimental animals. More than 25% of drugs used during the last 20 years are directly derived from plants, while the other 25% are chemically altered natural products. Still, only 5-15% of the approximately 250,000 higher plants have ever been investigated for bioactive compounds. The advantage of using such compounds for cancer treatment is their relatively non-toxic nature and availability in an ingestive form. An ideal phytochemical is one that possesses anti-tumor properties with minimal toxicity and has a defined mechanism of action. As compounds that target specific signaling pathways are identified, researchers can envisage novel therapeutic approaches as well as a better understanding of the pathways involved in disease progression. Here, we focus on 4 classes of natural anticancer drugs: methyltransferase inhibitors, DNA damaging/pro-oxidant drugs, HDAC inhibitors (HDACi), and mitotic disrupters, and we will focus on the mode of action for one promising example per group.

## INTRODUCTION

Cancers are characterized by the dysregulation of cell signaling pathways at multiple steps. However, most current anticancer therapies involve the modulation of a single target. The lack of safety and high cost of monotargeted therapies have encouraged alternative approaches. Both natural compounds, extracted from plants or animals, and synthetic compounds, derived from natural prototype structures, are now being used as cancer therapeutics and as chemopreventive compounds. In this report we will review four major classes of plant-derived anti-cancer drugs.

DNA methylation pattern is essential in development and can be altered in human tumors. Tumor cells are characterized by specific genetic and epigenetic changes that promote uncontrolled cellular proliferation. Based on the rationale that hypermethylation-induced gene silencing could be uncovered by gene demethylation and reactivation, many efforts have been put in the identification and characterization of inhibitors of DNA methylation as tools to treat cancer (1). Several studies suggested that green tea possess chemopreventive and therapeutic potential against tumor cells. Much of the anticancer and/or cancer chemopreventive effects of green tea are mediated by its most abundant catechin, epigallocatechin-3-gallate (EGCG). EGCG has been shown to possess strong anti-proliferative and anti-tumor effects both *in vitro* and in animal models. EGCG inhibited DNA methyltransferase activity with reactivation of epigenetically silenced tumor suppressor genes (1).

Chromatin acetylation is another major epigenetic modification that is regulated by the balanced action of histone acetyltransferases (HAT) and deacetylases (HDAC) (1). HDAC inhibitors (HDACi) reactivate epigenetically-silenced genes in cancer cells, triggering cell cycle arrest and apoptosis (2). HDACi can enhance the sensitivity to chemotherapy for cancers and inhibit angiogenesis. A number of natural and synthetic HDACi have shown an anti-proliferative activity on tumor cells. Recent evidence suggests that dietary constituents, such as the isothiocyanates found in cruciferous vegetables, can act as HDACi. Broccoli sprouts are a rich source of sulforaphane, an isothiocyanate that inhibits HDAC activity in human colon, prostate, and breast cancer cells (2, 3). Isoflavones have also been shown to possess a strong antioxidant activity and to inhibit oxidative DNA damage. Pomiferin, a prenylated isoflavonoid is isolated from *Maclura pomifera*. Pomiferin has been shown to inhibit the activity of HDAC enzyme. It also exhibited growth inhibitory activity on five human tumor cell lines including the HCT-15 colon tumor cell line (4).

Thymoquinone (TQ), the main bioactive component of the volatile oil of the black seed (*Nigella sativa*, Ranunculaceae family) (5), is a pleiotropic agent targeting multiple signaling pathways in many patho-physiological conditions. Recent studies have documented the cancer cell specific effects of TQ affecting multiple targets suggesting a promising role as an anticancer agent (6).

Drugs that inhibit microtubule dynamics represent some of the most effective anticancer medications. These drugs bind to tubulin, and are classified as microtubule stabilizers or destabilizers (7). The two major classes of antimitotic drugs used to treat cancer are the vinca alkaloids and the taxanes. Estramustine is another related drug that functions by binding to microtubules and MAPs and is used to treat prostate cancer. Vinca alkaloids were initially isolated from the pink periwinkle plant (*Catharantus roseus*; formerly vinca rosea Linn). The vinca alkaloids bind to β-tubulin near the GTP-binding site. Although the structures of the various vinca alkaloids vary only slightly, they have distinct niches as chemotherapeutic agents. Vincristine is most effective in treating leukemias, lymphomas and sarcomas. Vinblastine, which differs from vincristine only by substitution of a formyl for a methyl group, is effective in advanced testicular cancer, Hodgkin’s disease and lymphoma. Vinorelbine is currently used to treat non-small cell lung cancer as a single agent or in combination with cisplatin. Vindesine is undergoing clinical trials, primarily for treatment of acute lymphocytic leukemia. vinflunine, the newest member of the vinca alkaloid family is currently in clinical trials to test for activity against solid tumors (7). Another well-characterized drug-binding sites on tubulin/microtubules is the taxane-binding site. Taxanes are microtubule-targeting agents that bind to polymerized microtubules, stabilize the microtubule, and inhibit its disassembly leading ultimately to cell death by apoptosis. Paclitaxel (Taxol^®^, Bristol-Meyers Squibb) was originally derived from the bark of the Pacific yew tree but can now, like docetaxel, be partially synthesized from the precursor 10-deactylbaccatin III, derived from needles of the European yew (8).

Inhibitors of topoisomerase I and II are anticancer drugs active in a variety of haematological and solid tumours. The plant-derived camptothecins (irinotecan, topotecan) act as inhibitors of topoisomerase I; the plant-derived epopodophyllotoxins (etoposide and teniposide) and the microbial-derived anthracyclines (e.g. doxorubicin, epirubicin) act as inhibitors of topoisomerase II (9). Despite the numerous categories of the plant-derived anti-cancer drugs, this report reviews only 4 classes of natural anticancer drugs: methyltransferase inhibitors, HDAC inhibitors (HDACi), DNA damaging/pro-oxidant drugs and mitotic disrupters.

## DISCUSSION

### EGCG

EGCG has been shown to be an efficient scavenger of free radicals. There is evidence that the A-ring of EGCG may provide an antioxidant site (10, 11). On the other hand, studies have suggested that the cell-killing activity of tea phenols may be related to their pro-oxidant activity since in the presence of the H_2_O_2_ scavenger catalase, the EGCG-induced apoptosis was inhibited (12). Whereas EGCG has been shown to have strong antioxidant activity *in vitro*, such activity has been demonstrated only in some *in vivo* experiments (13). Among smokers, green tea consumption decreased oxidative DNA damage measured by lower urinary level of 8-hydroxydeoxyguanosine (14).

EGCG has been shown to exert antiproliferative effects by blocking the activation of transcription factors AP-1 and NF-kB by direct inhibition of specific kinases such as JNK (15, 16). EGCG can also inhibit cyclin-dependent kinases, leading to hypophosphorylated Rb protein form causing G0/G1 arrest (17).

EGCG has been reported to induce apoptosis in many cancer cell lines (18), including leukemia (19), stomach (20), pancreas (21), and breast (22, 23). EGCG sensitizes prostate carcinoma cells to TRAIL-mediated apoptosis (24), and it reduces telomerase activity in small-cell lung carcinoma (25). Caspase 3 activity seems to be required for green tea-induced apoptosis (26). Green tea has been shown to inhibit carcinogenesis induced by UV light and chemical carcinogens in rodents, as well as spontaneous tumorigenesis in wild-type and genetically modified mice (27-29). The drug was able to inhibit cancer growth and invasion in a xenograft mouse model with pancreatic cancer via up-regulation of caspase 3 activity and p21^WAF1^ expression (30).

EGCG was shown to have demethylating activity by inhibiting methyltransferases (31) and to elevate the transcription of tumor suppressor genes, an effect that can be further enhanced by the presence of HDACi (32).

Several studies have reported that EGCG inhibits the formation of new blood vessels by blocking VEGF expression in head and neck, breast, and colon cancer cells (33-35). In the TAMP mouse model, the expression of VEGF and matrix metalloproteases (36) and p-ERKs 1 and 2 (37) decreased when mice consumed green tea extract, and there were only low side-effects.

Many case-control studies have shown that subjects who consume large amounts of tea had a lower risk of gastric, esophageal (38), and breast cancer (39, 40). A recent encouraging study reported that among patients consuming 600mg green tea catechins daily within one year, there was a remarkable 90% reduction in the rate of high-grade-PIN-positive men developing prostate cancer (10). EGCG is currently tested in phase I pharmacokinetic study to determine its systemic availability after single oral dose administration (41). This clinical study is the first to show that chemicals in green tea can increase detoxification enzymes (glutathione S-transferases) in humans. Clinical trials of green tea products, especially in prostate cancer patients have yielded encouraging results (42).

Interestingly, investigating the pharmacogenetics of EGCG revealed that mice are very similar to humans in terms of enzymatic ability to conjugate tea catechins. Because the levels of tea consumption are lower than those used in animal cancer chemoprevention, the amount of the tea phenols that reaches the target tissues is a limiting factor. Furthermore, there is no doubt that the involvement of EGCG pro-oxidation may differ *in vivo* where anti-oxidative capacity is much higher and oxygen partial pressure is much lower than that in cell culture medium. Nevertheless, it is expected that cancer can be prevented by consuming moderate levels of tea especially for the oral cavity and the intestinal tract, and this concept has to be further tested in intervention human studies.

### Inhibitors of histone deacetylases pomiferin and sulforaphane

HDACi have been established as a potent and effective new means of therapy against various human cancers and recently the synthetic HDACi suberoylanilide hydroxamic acid (SAHA, vorinostat) has received FDA approval (43-46). Pomiferin, an isoflavone isolated from Osage orange (*Maclura pomifera*), and sulforaphane, an isothiocyanate isolated from broccoli, have recently been identified to posses anticancer properties via HDACi (47, 48).

Acetylation of lysine residues in histone molecules via HAT renders the chromatin into an open conformation, thus allowing gene transcription, while the opposing effects of HDAC lead to chromatin condensation (43, 44). The transcriptional control of HDACi target genes has been shown to be dependent on p53 and predominantly leads to the expression of the endogenous cyclin-dependent kinase inhibitor p21^cip1/waf^ (49, 50). Yet, a variety of non-histone proteins, e.g. p53, HIF-1α, Rb, β-catenin, HSP90, have also been shown to be substrates of histone deacetylases and therefore also account for the anti-tumor effects of these compounds (45, 51).

### Pomiferin

Pomiferin is a prenylated isoflavonoid from *Maclura pomifera*. Isoflavones have been shown to possess a strong antioxidant activity, i.e., to inhibit the production of reactive oxygen species (ROS) and to inhibit oxidative DNA damage. Pomiferin has first been investigated as a chemopreventive and antimicrobial agent (52, 53). Although the antioxidant effect opposes known HDACi effects like ROS formation, treatment of a cholangiocellular carcinoma cell line with pomiferin has shown pro-apoptotic effects via DNA fragmentation, as was previously described for other flavonoids, e.g. EGCG (see above) (54). In a proteomics approach, it was shown that pomiferin leads to downregulation of cytokeratins and to expression of known tumor-related proteins, e.g. S100A6 (54, 55). Recently, pomiferin has been demonstrated to inhibit HDAC enzyme activity at low micromolar concentrations (IC_50_ approx. 1 μM) and to inhibit growth of different human cancer cell lines, e.g. kidney, lung, prostate, breast or colon cancer, without affecting the growth of primary human hepatocytes (47).

### Sulforaphane

Sulforaphane is an isothiocyanate from various cruciferous vegetables like broccoli or its sprouts, and has originally been regarded as a chemopreventive dietary agent (56-58). The compound reaches high intracellular and plasma concentrations and has been shown to inhibit HDAC activity in human cancer cell lines (48). Sulforaphane was able to induce transcription of p21^cip1/waf1^ and to increase acetylation of histone H3 and H4, two established biomarkers for HDACi activity. Recently, sulforaphane has been shown to act as a direct inducer of human β-defensin-2 (HBD-2), an antimicrobial peptide that can be induced by HDAC inhibitors, in colonocytes suggesting a more direct role of sulforaphane in the treatment of colonic Crohn’s disease (2). Although localized hyperacetylation at the p21^cip1/waf1^ promoter has been shown, sulforaphane was also able to induce the transcription of p21^cip1/waf1^ in p53-deficient prostate cancer cells (59, 60). Higher doses of sulforaphane have been shown to induce oxidative stress and apoptosis (61-66). Interestingly, sulforaphane was able to induce apoptosis via the intrinsic bcl-2 dependent mitochondrial pathway as well as by the extrinsic TRAIL-dependent pathway (67, 68).

In xenograft models (e.g. prostate cancer, osteosarcoma), sulforaphane leads to growth retardation, inhibition of HDAC activity, and increase in acetylated H3 and H4 levels (64, 69). Furthermore, an antiangiogenic effect has been reported *in vitro* (70). Longterm treatment with this compound has lead to decreased tumor formation in the APCmin mouse model, which was also paralleled by transcriptional regulation of p21^cip1/waf1^ and bax (71). In mouse preclinical models, sulforaphane inhibited HDAC activity and induced histone hyperacetylation coincident with tumor suppression. Inhibition of HDAC activity was also observed in circulating peripheral blood mononuclear cells obtained from people who consumed a single serving of broccoli sprouts (3).

In humans, oral intake of broccoli leads to HDACi in PBMCs (determined by HDAC activity and acetylation of H3 and H4) already after 3h and returned to normal levels after 24h (69). Importantly, no severe adverse events or changes in laboratory parameters were observed in a clinical trial (72). Sulforaphane was also detectable in breast tissues after single oral administration, indicating good pharmacologic properties also in humans (73).

As the overall efficacy of plant-derived HDACi is comparably lower than that of synthetic or fungal HDACi (e.g. Trichostatin A, SAHA), their potency is seen in chemoprevention of cancer diseases due to their anti-oxidant effect as these compounds are readily available by dietary intake. So far, no further data from controlled clinical trials with purified sulforaphane is available, but several trials are currently in preparation or already recruiting to investigate the effect of this promising compound in human cancer diseases (see www.clinicaltrials.gov for details).

The clinical experience with hydroxamic acid (e.g. Vorinostat (SAHA), Panobinostat (LBH589), Belinostat (PDX101) or benzamide HDACi (e.g. MS275) proved a good tolerability of HDACi. Adverse effects were usually manageable and consisted of fatigue, nausea, and other gastrointestinal symptoms, while hematological symptoms are rare and not dose-limiting. In earlier studies, cardiac QTc prolongation was repeatedly reported for different HDACi and considered as the major drawback for the future development (74-76). Yet, these findings were not confirmed for modern and orally available HDACi, such as Panobinostat, which rarely displayed cardiac toxicity (77).

### Thymoquinone

TQ is the bioactive constituent of the volatile oil of black seed (*Nigella sativa*). Black seeds have been used for thousands of years for medical purposes in Middle Eastern and Asian countries, and for this reason, it is named “the blessed seed”. TQ has been found to be the main compound responsible for the biological effects of the seeds (78).

TQ has been reported to have potent anticancer and superoxide anion scavenging abilities in animal models and cell culture systems (79). Under physiological conditions and in human erythrocytes, TQ directly interacted with glutathione and NADH to reduce the ferryl forms of met-hemoglobin and met-myoglobin to their oxidized forms, thus leading to the recovery of hemoglobin and myoglobin from oxidative stress (80). In other studies, TQ was shown to act as an antioxidant and inhibited iron-dependent microsomal lipid peroxidation (81), cardiotoxicity induced by doxorubin in rats (82, 83), and ifosfamide-induced damage in kidney (84). It also prevented carbon tetrachloride-induced hepatic injury (85, 86). In all mentioned models, TQ reduced drug toxicity and caused improvements in the drug’s anticancer activity. On the other hand, there are studies reporting that the anticancer potential of TQ is related to its pro-oxidant activities. In human colon cancer cells and in isolated rat liver mitochondria, TQ induced a significant release of reactive oxygen species (ROS) and inhibited the activity of aconitase, an enzyme sensitive to superoxide anion generation (87).

One of the most promising effects of TQ is that it exhibits high cancer specificity and low toxicity to normal cells. This has been observed in prostate cancer (88), colon cancer (89, 90), canine osteosarcoma (90), and skin cancer (91). Many multidrug-resistant variants of human pancreatic adenocarcinoma, uterine sarcoma, and leukemia were found to be sensitive to TQ (92).

The mechanisms of TQ anticancer action in cells range from the induction of G0/G1 arrest in colon, canine osteosarcoma and mouse papilloma cells (90, 91-94), to G1/S phase arrest in prostate (88), and G2/M arrest in skin (91). TQ-induced growth arrest is linked to the increased levels of the cyclin-dependent kinase (CDK) inhibitors p16^INK4^, p21^WAF1^, and p27^Kip1^ (88, 89, 91), downregulation of androgen receptor, transcription factor E2F-1, and its positive regulator p-Rb (89). The black seed oil and its ethyl extract have shown anti-tumor properties in a variety of cell lines such as ICO1, Vero cells and BSR cell lines. TQ and its synthetic derivatives have been shown to inhibit the function of the serine/threonine kinase Polo-like kinase 1 (Plk1 PBD) *in vitro*, and cause Plk1 mislocalization, chromosome congression defects, mitotic arrest, and apoptosis in HeLa cells. These results provide a great potential into the development of synthetic derivatives of TQ as anticancer agents (6).

TQ induces apoptosis through modulation of multiple targets and hence is a promising phytochemical that could be useful for the killing of many types of cancer cells. These results are also supported by reports in prostate and other cancer cells (6).

TQ induces apoptosis in cells by p53-dependent (89) and p53-independent pathways (92), and drug-induced apoptosis is associated with the activation of caspases (92-94), increases in p53 expression (44), up-regulation of pro-apoptotic Bax and downregulation of anti-apoptotic Bcl-2 (87, 91), and decrease in cyclins B1 and D1 (91). In SW-626 human colon cancer cells, TQ induced major cellular damage and severely impaired the normal cellular metabolism, effects that were comparable to those triggered by 5-flourouracil, a colon cancer chemotherapeutic agent (95). Moreover, recent studies have shown that NF-κB is a legitimate target of TQ which was associated with cell growth inhibition and induction of apoptosis in cancer cells (6).

*In vivo*, TQ inhibited the growth of prostate (89) and colon (87) tumors implanted in nude mice with no noticeable side effects. In colon xenografts, growth inhibition by TQ was not due to decreased proliferation but rather to the significant induction of apoptosis (87). However, in androgen-independent prostate tumor xenografts, the suppression of tumor growth was associated with a marked decrease in E2F-1 and induction of massive apoptosis (88). TQ blocked angiogenesis *in vitro* and *in vivo*, prevented tumor angiogenesis in a xenograft human prostate cancer (PC3) model in mouse and inhibited human prostate tumor growth with almost no side effects (5). In mouse, injection of the essential oil into the tumor site significantly inhibited solid tumor development and the incidence of liver metastasis, thus improving mouse survival. These results indicate that the anti-tumor activity or cell growth inhibition could in part be due to the effect of TQ on cell cycle (6).

Despite the promising anticancer effects of TQ, there has been no attempt to clinically test the drug in humans. This is mainly due to the lack of pharmacological data on the molecule, namely its absorption, distribution, metabolism, and excretion. One reason could be the difficulty of analyzing the compound *in vivo.* TQ is highly reactive with thiol compounds and interacts directly with glutathione (80), an enzyme common in the human system. Our recent attempts to detect and quantify TQ in blood samples of rats injected with the drug have revealed major challenges in determining its pharmacokinetic and dynamic properties. The drug readily complexes with enzymes, interacts with cellular membranes, and cannot be detected in free form, and the latter is necessary for subsequent quantification and determination of its kinetics. Further challenges for translating this drug to the clinic include its low solubility in aqueous solutions. TQ is soluble in methanol, which is toxic if ingested by humans. Future studies in our laboratories will investigate newly-synthesized more soluble derivatives that are as potent as the parent molecule.

### Microtubule Disruptors: Paclitaxel

No bioactive compound discovered over the last 30 years has attracted more public attention than paclitaxel (96). Paclitaxel is a complex taxane diterpene isolated from the bark of *Taxus brevifolia* (97). The cytotoxic activity of the bark extract was first reported in 1963, utilizing KB cytotoxicity assay. Subsequently, Paclitaxel´s *in vivo* activity against mouse leukemia was discovered in1966 (98), and its structure was described in 1971 (99).

Microtubule-targeting drugs inhibit the metaphase anaphase transition through suppressing spindle microtubule dynamics, which block mitosis and induce apoptosis (100). Microtubule stabilizing agents (MSA) is a class of these drugs that includes taxanes (paclitaxel and docetaxel), epothilones A and B, discodermolide, eleutherobin (100), and monastrol (101, 102). These agents stabilize microtubules by binding to polymeric tubulin, thus preventing disassembly (103, 104). Paclitaxel causes polymerization and stabilization of microtubules in tumor cells, thereby inhibiting cell replication through disruption of normal mitotic spindle formation (105). Therefore, cells treated with paclitaxel are unable to proceed normally through the cell cycle and arrest in G2/M phase (106). This halt of the cell cycle at mitosis has been considered the cause of paclitaxel-induced cytotoxicity (107).

Paclitaxel triggers apoptosis by caspase-dependent and independent pathways (108) that regulate the expression of apoptosis-related proteins such as Bim, Bcl-2, Bad, Bcl-XL, p21^WAF-1/CIP-1^, tumor necrosis factor-α (TNF-α) receptor 1 (TNFR1), and the TRAIL receptors DR4 and DR5 (107, 109-113). Recently, it has been suggested that paclitaxel changes the translational machinery that occurs during apoptosis. Paclitaxel inhibits the translational machinery by increasing elongation factor eEF2 phosphorylation. In addition to its ability to trigger various signal transduction pathways, including JNK, p38^MAPK^, and ERK, paclitaxel has also been reported to promote the activation of JNK/SAPK through Ras and ASK1 pathways (114). JNK phosphorylates and inactivates Bcl-2 at the G2M phase of the cell cycle as demonstrated by the inhibition of paclitaxel-induced phosphorylation of Bcl-2 using dominant negative mutants of JNK and ASK1 (115). Following paclitaxel treatment, when mitotic arrest and mitotic slippage occur, survivin is downregulated (116) and Aurora B is inactivated (117), enabling apoptosis to occur in G1. Overexpression of survivin has been shown to be associated with increased resistance to paclitaxel-induced cell death (116). On the other hand, inhibition of survivin by mitotic inhibitors such as oxaliplatin, increases paclitaxel-induced apoptosis and cell death in colonic carcinoma cells (117). Paclitaxel and cisplatin are widely used anticancer agents for treatment of non-small cell lung cancer (118).

Paclitaxel treatment induces the expression of IL-8 in ovarian and in non-small lung cancer cell lines (119, 120) as well as in patients (121, 122). In addition, Paclitaxel up-regulates IL-6 in cell lines and patients (123). NF-kB-dependent transcription of COX-2 is upregulated in the presence of Paclitaxel (124). A recent study has documented increased levels of COX-2 in specimens taken from patients undergoing Paclitaxel treatment for non-small cell lung carcinoma, demonstrating the relevance of the clinical effect (125).

A combination therapy of phase II clinical trials with taxane and celecoxib, a COX-2 inhibitor, has yielded mixed results, making further investigation necessary (126).

Paclitaxel completed many clinical trials from 1982-2003 (98, 127-133). In the phase III MDACC trial (134), a slight increase in disease-free survival (DFS) and overall survival (OS) was observed in FAC followed by paclitaxel (paclitaxel) (P) (FAC-P) arms compared to FAC alone. The first large prospective trial to examine the addition of paclitaxel to an anthracycline-based regime in node-positive women was undertaken by the CALGB 9344 trial (135). The addition of paclitaxel significantly improved DFS 70% vs. 65% and OS 80% vs. 77%. In the NSABP B28 trial (136), the addition of paclitaxel to adjuvant anthracycline therapy improved the 5-year DFS regardless of tumor grade, histological type, patient´s age, or number of positive lymph nodes, although there was no improvement in 5-year OS. In the Cancer and Leukemia Group B 9741 trial, doxorubicin (A), cyclophosphamide (C), and paclitaxel (P) administration was compared in sequential versus concurrent regimes in the setting of either conventional administration three times weekly or dose-dense administration twice weekly. Moreover, 2005 women with node-positive metastatic breast cancer were randomly assigned to one of the four treatment arms illustrated in (137, 138); significant improvements were seen in DFS, OS, relapse risk, and mortality risk with dose-dense scheduling. In addition to other micellar formulations in preclinical developments, the paclitaxel-based nanoparticulates (NK105) have recently been advanced into clinical trials (138-140).

Data from clinical trials incorporating trastuzumab with paclitaxel in operable or metastatic breast cancer have shown increased synergy between taxanes and trastuzumab when combined together (141-143).

After recognizing paclitaxel’s activity against breast cancer in 1991, the fear of short supply has emerged. Bristol-Myers Squibb is currently producing paclitaxel from plant tissue cultures in Germany (98, 144). In addition, many marine-based natural products with paclitaxel-like activity have been identified recently (145-148), and some of them are in Phase I clinical trials (149).

Resistance to chemotherapeutic agents is a very challenging and complex phenomenon, orchestrated by a number of complex mechanisms in a single cell (145). Paclitaxel is susceptible to several mechanisms of drug resistance, most importantly expulsion from the cell by the multi-drug resistance transporter P-glycoprotein (P-gp). This precludes the use of taxanes against blood-borne cancers, which commonly express P-gp. Along with other side-effects, thrombosis, bradycardia, heart block and hypotension have been reported to be associated with paclitaxel treatment (150-152).

## CONCLUSION

**Figure 1:**
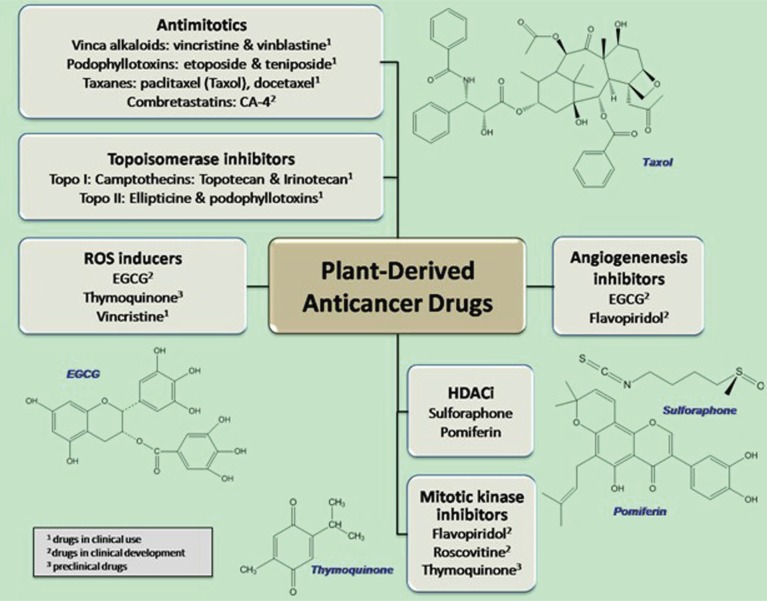
Selected groups of plant-based anticancer drugs. Some drugs may execute their therapeutic and/or chemoprotective functions through multiple pathways. EGCG is well-known for its ROS-related activity; it may also function as inhibitor of DNA methylation and angiogenesis. Thymoquinone is a ROS inducer as well as a potent mitotic kinase inhibitor.

It is apparent that at present, drug-based therapeutic strategies will predominate in the 21^st^ century. Thus, the discovery of new drugs effective against resistant tumors is an important and necessary strategy in improving chemotherapy. Natural drugs have found direct medical application as drug entities, but they also serve as chemical models or templates for the design, synthesis, and semisynthesis of novel substances, such as paclitaxel (Taxol®), vincristine (Oncovin®) and camptothecin, in the treatment of human cancer (Figure 1). Although there are some new approaches to drug discovery, such as a combination of chemistry and computer-based molecular modeling design, none of them can replace the important role of natural products in drug discovery and development.
